# Severe myocardial ischemia detected in routine oncological positron emission tomography/computed tomography with ^18^F-fluorodeoxyglucose

**DOI:** 10.1093/eurheartj/ehad208

**Published:** 2023-04-08

**Authors:** Martin T Freitag, Philip Haaf, Michael J Zellweger

**Affiliations:** Division of Nuclear Medicine, University Hospital of Basel, University of Basel, Petersgraben 4, 4031 Basel, Kanton Basel-Stadt, Switzerland; Clinic of Cardiology, University Hospital of Basel, University of Basel, Petersgraben 4, 4031 Basel, Kanton Basel-Stadt, Switzerland; Clinic of Cardiology, University Hospital of Basel, University of Basel, Petersgraben 4, 4031 Basel, Kanton Basel-Stadt, Switzerland

A 69-year-old man underwent positron emission tomography/computed tomography with ^18^F-fluorodeoxyglucose (^18^F-FDG-PET/CT) for staging of recurrent seminoma. He had no prior known cardiac disease but stable angina pectoris. The patient fastened as per protocol for 6 h with blood glucose 5.3 mmol/L prior to injection of ^18^F-FDG. A strong ^18^F-FDG-uptake was observed only in the apex of the heart, whereas the rest of the myocardium showed incidental suppressed myocardial FDG-uptake. Without specific preparation, this condition is less common in routine ^18^F-FDG-PET/CT and also termed ‘lipid-shift’ of cardiac metabolism (*Panel A*). The CT images showed calcified coronary arteries. He was referred for a ^82^rubidium-PET/CT scan, which revealed severe ischemia in the territory of the left anterior descending artery (LAD) matching the region of the ^18^F-FDG-uptake (*Panel B*). Coronary angiography confirmed occlusion of the apical LAD (*Panel C*) with retrograde perfusion by right coronary artery, and the patient successfully underwent non-invasive coronary revascularization.

**Figure 1:**
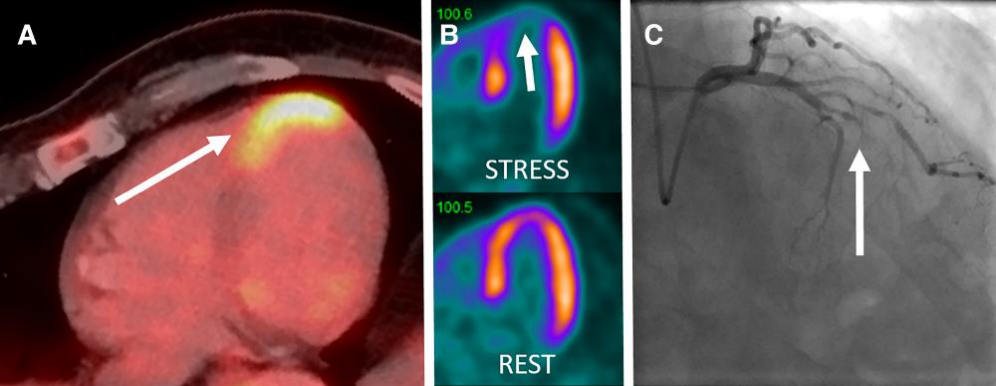
(*A*) Positron emission tomography/computed tomography scan with ^18^F-fluorodeoxyglucose with increased uptake in the apex (white arrow) while the rest of the left myocardium is incidentally suppressed (‘lipid shift’). (*B*) Afterwards, stress-/rest positron emission tomography/computed tomography scan with ^82^rubidium confirms severe ischemia in the apex visible in the long axis view (white arrow). Summed difference stress score was 10 (severe ischemia). (*C*) Coronary angiography confirms occlusion of the left anterior descending (white arrow).

Chronic ischemia results in metabolic adaptations allowing the cardiomyocytes to survive in a hibernating, low contractility state. During this hibernation process, de-differentiation of cardiomyocytes may occur and induces a reliance on glucose for energy provision—similar to the fetal heart—in order to improve cell survival.^1,2^

The case highlights that severe ischemia and hibernating myocardium may be detected in routine ^18^F-FDG-PET/CT scans without stress testing under specific circumstances (incidental ‘lipid-shift’ and coronary pattern of increased ^18^F-FDG-uptake), which is of paramount importance for patients undergoing oncologic PET scans with unknown cardiac disease.^3,4^

The data underlying this article will be shared on reasonable request to the corresponding author.

The authors declare no conflict of interest related to this manuscript.
